# Arm movement adaptation to concurrent pain constraints

**DOI:** 10.1038/s41598-021-86173-7

**Published:** 2021-03-24

**Authors:** Johannes Kühn, Carlo Bagnato, Etienne Burdet, Sami Haddadin

**Affiliations:** 1 Chair of Robotics and System Intelligence, Munich School of Robotics and Machine Intelligence, Technical University Munich, 80797 Munich, Germany; 2 Department of Bioengineering, Imperial College London, London, SW7 2AZ UK; 3 FRANKA EMIKA GmbH, Frei-Otto-Straße 20, 80797 Munich, Germany

**Keywords:** Human behaviour, Cognitive neuroscience, Motor control

## Abstract

How do humans coordinate their movements in order to avoid pain? This paper investigates a motor task in the presence of concurrent potential pain sources: the arm must be withdrawn to avoid a slap on the hand while avoiding an elbow obstacle with an electrical noxious stimulation. The results show that our subjects learned to control the hand retraction movement in order to avoid the potential pain. Subject-specific motor strategies were used to modify the joint movement coordination to avoid hitting the obstacle with the elbow at the cost of increasing the risk of hand slap. Furthermore, they used a conservative strategy as if assuming an obstacle in 100% of the trials.

## Introduction

**Figure 1:**
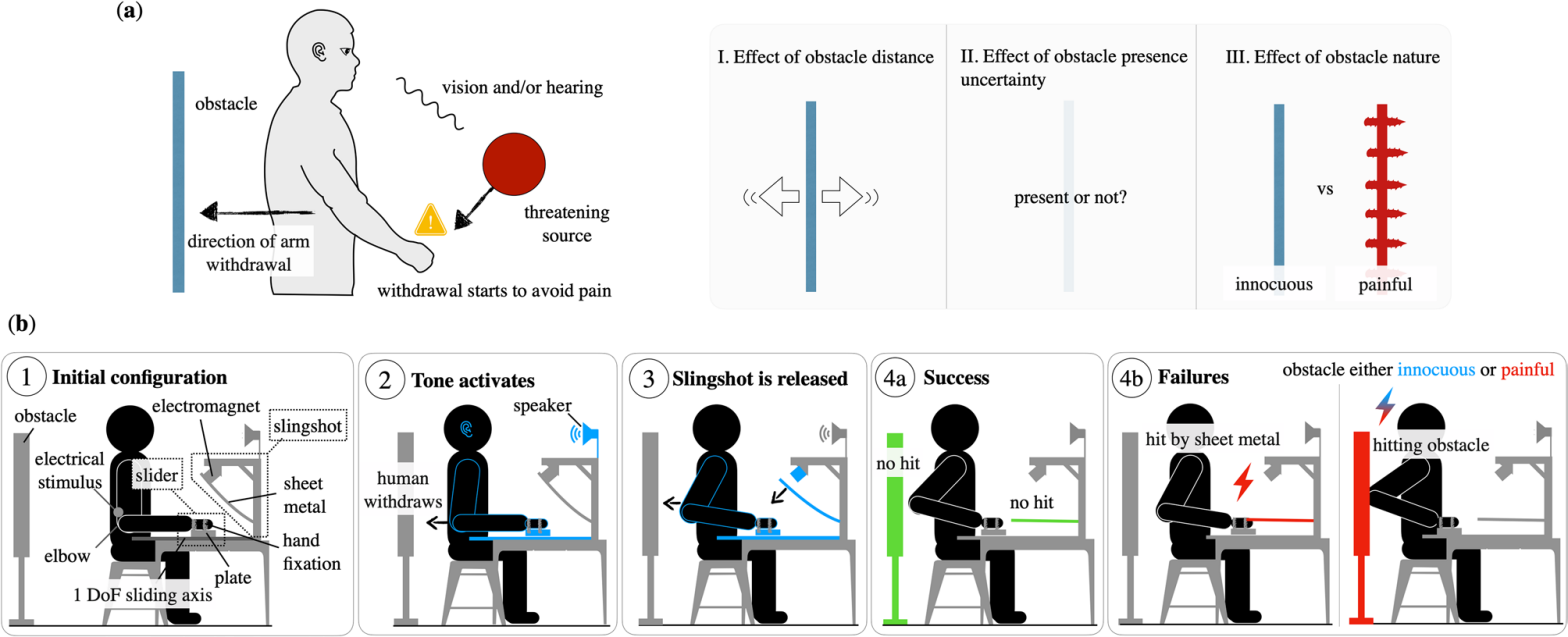
(**a**) Scenario under study. (**b**) Sketch of experimental setup and sequence.

Pain helps humans to avoid injuries by signaling the immediate threat and triggering relevant motor responses, e.g. the withdrawal reflex. While the withdrawal reflex is defined as an automatic retraction of an extremity from a noxious stimulus such as heat or pain, it is also common to initiate withdrawal actions prior to any physical stimulation, for instance when we visually spot the threatening event. This is what would happen when our hand is about to get slammed into the heavy cutlery drawer, as our cheerful son suddenly pushes it with force. To prevent pain and injury, we would retract the hand from inside the drawer as fast as possible. Additionally, we may have to consider several constraints and pain sources simultaneously. Imagine that our son is along the direction of withdrawal at that point in time. The motor control dilemma arises on how to preserve physical integrity and at the same time modulate the instinctive withdrawal of the hand in such a way that the kid is not hit by the retraction of the arm. How do humans deal with such constraints during motor tasks? Corresponding to this scenario (Fig. 1a):

I. Do we—and, if yes, how do we—use the mechanical redundancy of our upper limb when a constraint (i.e. an obstacle behind us) is introduced that limits the room for withdrawal? (Effect of obstacle distance)
II. Does our motor strategy change when the environmental conditions are subject to uncertainty? Specifically, if an obstacle may or may not be present behind us, do we adopt a conservative strategy and plan the movement according to the worst-case scenario or do we rather take risks? (Effect of obstacle presence uncertainty)
III. How does the introduction of pain—as a consequence of the possible impact with the obstacle—affect the way we avoid the obstacle when withdrawing our arm? (Effect of obstacle nature)

To address these questions, we have developed a paradigm where sudden withdrawal of the upper limb is elicited by means of a threatening mechanical source at the level of the hand while they could hit an obstacle on the back with the elbow. To escape pain at the hand, subjects necessarily had to retract the arm while an obstacle impedes the path of the elbow during hand retraction. We compared their withdrawal actions across different geometric and pain conditions.

## Results

### Experiment overview

Each subject was seated on a chair with the hand fixed on a one degree-of-freedom (DoF) *slider* mechanism as shown in Fig. 1b $\textcircled {1}$. Above the hand was a preloaded *slingshot*, a flexible sheet metal retained by an electromagnet. An acoustic signal—aired 500 ms before the electromagnet was switched off (Fig. 1b $\textcircled {2}$)—was used to warn the subject, who was thus prompted to retract the hand and so avoid the impact with the sheet metal (Fig. 1b $\textcircled {3}$). An obstacle was placed behind the elbow. The obstacle was *innocuous* for subjects of a first group, and *painful* for the second subjects’ group with an electrical stimulation triggered at the elbow as soon as the panel was touched. The task’s goal for all subjects (Fig. 1b $\textcircled {4}$) was to withdraw the hand (1) to escape the mechanical pain threat by the slingshot mechanism (2) while avoiding colliding the obstacle with the elbow. For both groups of subjects, the start condition of each trial was one of $\{away, midway, close, close_{50\%}\}$, corresponding to the distance between the elbow position and the obstacle. The *close* and *midway* conditions are used to investigate how subjects deal with a challenging versus moderate difficulty level, relative to the *away* condition without obstacle. In the $close _{50\%}$ condition, participants were informed that there would be an obstacle placed at the *close* distance in 50% of the cases. A detailed description of the experiment, definitions of the measures used and data analysis can be found in “Methods” section.

### Preliminary inspection of data

**Figure 2:**
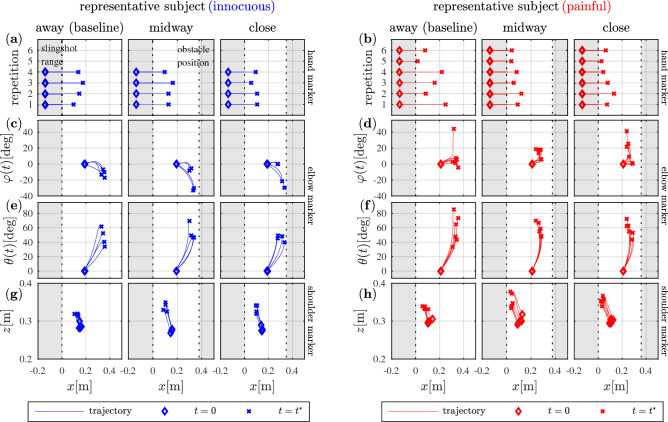
Raw data of representative subjects from each group. (**a**,**b**) Trajectories of hand marker (*x*-coordinate) for all trials of one representative subject from each group. (**c**,**d**) Lateral elbow displacement $\varphi (t)$ over *x*-coordinate values of the elbow marker for all trials of one representative subject from each group. (**e**,**f**) Upward elbow displacement $\theta (t)$ over *x*-coordinate values of the elbow marker for all trials of one representative subject from each group. (**g**,**h**) Cartesian trajectories of shoulder marker in *xz*-plane. The definition of $\varphi (t)$, $\theta (t)$ and the right-handed coordinate system are depicted in Fig. 6c.

Figure 2 shows raw data from representative subjects of each group.

Visual inspection of trajectories reveals that the hand retracts less when the obstacle gets closer (Fig. 2a). By retracting the hand, subjects bend their elbows sideways to the right of the longitudinal axis of the hand, to the left of or along it (Fig. 2c). Reviewing all subjects individually, we noted that subjects who bend their elbow to the inside ($\varphi (t)<0$) retract the hand less, while other subjects opt for longer spans of hand retraction, but bend their elbow to the outside ($\varphi (t)>0$) and with more displacement. When the obstacle is introduced, the lateral movement of the elbow seems to increase. As the elbow moves sideways during hand retraction, it also shifts upwards (Fig. 2e). Such combined movement is more accentuated for the subjects who bend their elbow to the outside (the ones with greater hand retraction and lateral elbow displacement). During the withdrawal action, the shoulder moves upwards and forward (Fig. 2g). Comparing the representative subjects from the two groups, the hand seems to retract less when subjects are presented with the painful obstacle, in contrast to the innocuous one (Fig. 2a vs. b). With respect to the lateral elbow displacement $\varphi (t)$, no systematic differences between the *painful* and *innocuous* groups could be seen (Fig. 2c vs. d). Upward elbow displacement $\theta (t)$ versus hand retraction appears to be steeper for the *painful* group (Fig. 2e vs. f). With respect to the shoulder movement, no remarkable differences could be seen between the *painful* and *innocuous* groups (Fig. 2g vs. h).

### Effect of obstacle distance

Placing an obstacle closer leads to more hits with the obstacle (Table 1). This shows that the task was challenging enough to represent limit cases. Although more task errors were recorded when the obstacle distance was reduced, the percentage of failures collected in Table 1 suggests that the task, although challenging, was achievable in most of the occurrences.

**Table 1:** Failure ratios *f* (3).

Condition	Obstacle type	*f* [%]			
		Obstacle only	Slingshot only	Both	Sum
Away	Innocuous	–	0	–	0
	Painful	–	1.39	–	1.39
Midway	Innocuous	9.09	4.55	0	13.64
	Painful	2.78	4.17	1.39	8.34
Close	Innocuous	25.00	2.27	2.27	29.54
	Painful	8.33	15.28	0	23.61
Close$_{50\%}$	Innocuous	6.82^a^	4.55	0^a^	11.37^a^
	Painful	5.56^a^	15.28	0^a^	20.84^a^

^a^Note that, in the $close _{50\%}$ condition, errors were only possible when the obstacle was present.

Results exhibit no significant differences for reaction times (Table 2) among the *away*, *midway* and *close* conditions tested.

**Table 2:** Reaction time $\Delta t_{\mathrm{r}}$ (2).

	Innocuous [ms]	Painful [ms]
Away	$214.2\pm 20.8$	$214.8\pm 40.9$
Midway	$224.4\pm 30.9$	$218.9\pm 39.2$
Close	$210.9\pm 25.8$	$226.8\pm 53.6$
Close$_{50\%}$	$209.3\pm 22.2$	$229.4\pm 51.8$

**Figure 3:**
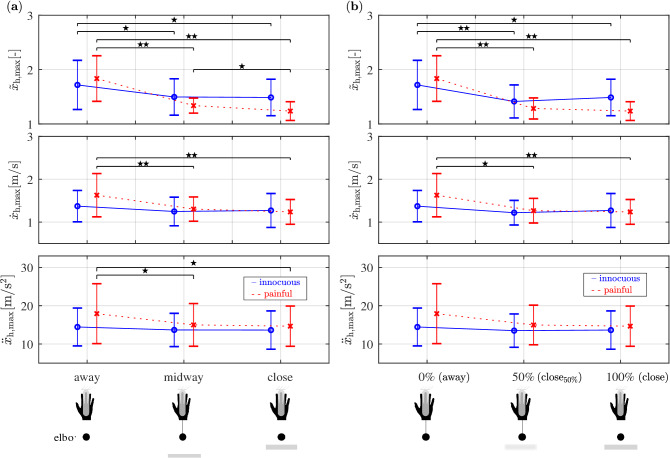
(**a**) Maximum hand retraction $\tilde{x}_{\mathrm{h},\mathrm{max}}$(1), $\dot{x}_{\mathrm{h},\mathrm{max}}$ and $\ddot{x}_{\mathrm{h},\mathrm{max}}$ over varying obstacle distances $\{away,midway,close\}$, for both *innocuous* (blue) and *painful* (red) groups. The closer the wall, the less the hand retracts. This effect is more remarkable for obstacle type *painful* (red). Only when the painful obstacle is presented (red), subjects slow down speed and decrease acceleration of hand retraction. $\star =p<0.05$, $\star \star =p<0.01$; corrected. (**b**) Maximum hand retraction $\tilde{x}_{\mathrm{h},\mathrm{max}}$, $\dot{x}_{\mathrm{h},\mathrm{max}}$ and $\ddot{x}_{\mathrm{h},\mathrm{max}}$ over varying probabilities of obstacle presence $\{0\%,50\%,100\%\}$, for both *innocuous* (blue) *painful* (red) groups. Probabilities were made explicit to subjects prior to the start of each trial. In the trials when the obstacle was present, it was positioned in the *close* configuration. No differences were found between the 50% (i.e. condition $close _{50\%}$, when the obstacle was present in half of the trials) and 100% (i.e. *close* condition) probabilities. $\star =p<0.05$, $\star \star =p<0.01$; corrected.

**Figure 4:**
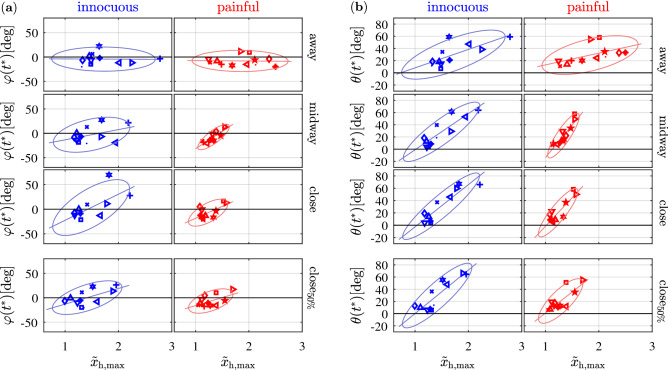
The figure shows values of sideways displacement $\varphi (t^*)$ of the elbow (**a**) and values of upward displacement $\theta (t^*)$ of the elbow (**b**) at the point of maximum hand retraction $\tilde{x}_{\mathrm{h},\mathrm{max}}$ (1). Each symbol represents the mean of such dyad for one individual subject. $\varphi (t^*)$ distributes evenly along the axis of hand retraction, and subjects seem to stick to one specific strategy (left, right or straight) over the different conditions, with only one exception (see blue triangle for innocuous). Ovals denote confidence ellipses with confidence interval 90%. Introducing a painful obstacle (red) reduces the area of the confidence ellipses. Solid lines denote the fitted linear models. Their slope increases as obstacles are introduced.

**Figure 5:**
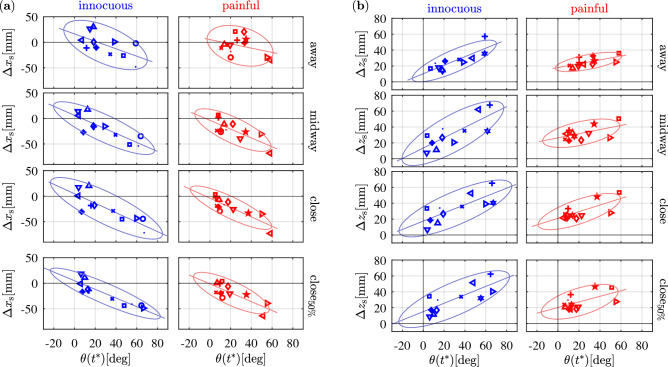
The figure shows mean values of shoulder displacement in the *x*-direction $\Delta x_{\mathrm{s}}$ (**a**) and in the *z*-direction $\Delta z_{\mathrm{s}}$ (**b**) at the time $t^*$ over the elbow upward displacement $\theta (t^*)$. Each symbol represents the mean of such dyad for one individual subject. Ovals denote confidence ellipses with confidence interval 90%. Introducing a painful obstacle (red) reduces the area of the confidence ellipses. Solid lines denote the fitted linear models. In both cases, slopes do not change over conditions.

**Table 3:** Results of linear regression in Figs. 4 and 5.

Condition	Fig. 4a				Fig. 4b				Fig. 5a				Fig. 5b			
	Innocuous		Painful		Innocuous		Painful		Innocuous		Painful		Innocuous		Painful	
	$R^2$	Slope	$R^2$	Slope	$R^2$	Slope	$R^2$	Slope	$R^2$	Slope	$R^2$	Slope	$R^2$	Slope	$R^2$	Slope
away	0.00	$-\,0.48$	0.00	$-\,0.81$	0.53	30.2	0.30	19.2	0.37	$-\,0.73$	0.11	$-\,0.42$	0.68	0.54	0.40	0.24
midway	0.18	21.0	0.67	71.6	0.76	59.3	0.74	106.9	0.70	$-\,0.88$	0.58	$-\,0.86$	0.62	0.65	0.29	0.26
close	0.45	53.0	0.43	48.4	0.84	68.5	0.76	92.7	0.69	$-\,0.93$	0.72	$-\,0.92$	0.62	0.52	0.52	0.44
close$_{50\%}$	0.37	32.2	0.30	32.9	0.77	71.4	0.62	69.0	0.81	$-\,0.85$	0.61	$-\,0.87$	0.62	0.53	0.35	0.35

The hand retracts less when the obstacle gets closer, as showed by the significant differences of $\tilde{x}_{\mathrm{h},\mathrm{max}}$ (Fig. 3a). The linear mixed-effects analysis showed significant effects of the interaction between distances $\{away,midway,close\}$ and obstacle types $\{innocuous,painful\}$, on $\tilde{x}_{\mathrm{h},\mathrm{max}}$ ($\chi ^2(1)=5.70, p<0.05$), $\dot{x}_{\mathrm{h},\mathrm{max}}$ ($\chi ^2(1)=7.04, p<0.01$), and $\ddot{x}_{\mathrm{h},\mathrm{max}}$ ($\chi ^2(1)=4.96, p<0.05$). Paired t-tests applied on the mean values of $\tilde{x}_{\mathrm{h},\mathrm{max}}$, $\dot{x}_{\mathrm{h},\mathrm{max}}$ and $\ddot{x}_{\mathrm{h},\mathrm{max}}$ revealed significant differences (Fig. 3a) of $\tilde{x}_{\mathrm{h},\mathrm{max}}$ for the obstacle type *innocuous*: $\tilde{x}_{\mathrm{h},\mathrm{max}}$ decreases from condition *away* to *midway* ($p<0.05$), and also from *away* to *close* ($p<0.05$). No significant differences were found on $\dot{x}_{\mathrm{h},\mathrm{max}}$ and $\ddot{x}_{\mathrm{h},\mathrm{max}}$. Within group *painful* paired t-tests revealed significant differences (Fig. 3a): $\tilde{x}_{\mathrm{h},\mathrm{max}}$ decreases from condition *away* to *midway* ($p<0.01$), from *away* to *close* ($p<0.01$), and from *midway* to *close* ($p<0.05$). Significant differences were found concerning $\dot{x}_{\mathrm{h},\mathrm{max}}$ that decreases from condition *away* to *midway* ($p<0.01$), and from *away* to *close* ($p<0.01$). Furthermore, significant differences were found concerning $\ddot{x}_{\mathrm{h},\mathrm{max}}$ that decreases from condition *away* to *midway* ($p<0.05$), and from *away* to *close* ($p<0.05$).

The linear mixed-effects analysis showed no significant differences for $\varphi (t^*)$ and $\theta (t^*)$. However, values of mean $\tilde{x}_{\mathrm{h},\mathrm{max}}$ for each subject, at all conditions, were visually inspected by plotting them with $\varphi (t^*)$ and $\theta (t^*)$, respectively (Fig. 4). Different symbols were used for individual subjects to enable tracking of any change in motor behavior, as the conditions of the experiment vary. By retracting the hand, subjects bend their elbow sideways (Fig. 4a), either to the right of, to the left of, or along the longitudinal axis of the hand, in any case sticking to one of such strategies over the different distance conditions they are presented with. The linear regression of Fig. 4a (solid lines) exhibit slopes steeper as the obstacle distance decreases, indicating that a certain value of hand retraction corresponds to a bigger lateral displacement of the elbow (see Table 3 for the $R^2$ and slope values of linear regression). This behavior is coherent with task requirements, as subjects tend to keep away from collision by bending the elbow more when the obstacle is closer. The same tendency is observed for $\theta (t^*)$. As the elbow moves sideways during hand retraction, it also shifts upwards (Fig. 4b), with such effect becoming more emphasized as the obstacle gets closer.

To further analyze the kinematic chain of the upper limb, the shoulder front and upwards movements were displayed against the values representing the elbow upward movements. Although the linear mixed-effects analysis showed no significant differences for $\Delta x_{\mathrm{s}}$ and $\Delta z_{\mathrm{s}}$, we see in Fig. 5a,b that the higher $\theta (t^*)$, the more forward the shoulder moves. Similarly, the higher $\theta (t^*)$, the more upwards the shoulder moves.

### Effect of obstacle presence uncertainty

While no significant differences were found with the analysis of reaction times (Table 2), the results of Table 1 reveal that the failure ratio in the $close _{50\%}$ condition matches the one recorded for the *close* condition. While subjects consistently escape impact from the slingshot stimulation at their hand when the *away* condition is presented, more errors occur when they know that there is an obstacle behind them (*close* condition), with the ratio of failure registered for the $close _{50\%}$ condition comparable to the one in the *close* condition. The same reasoning cannot be applied fully to study the failure ratios regarding the collisions with the obstacle, as no obstacle is present by definition in the *away* condition (0%), and the obstacle is present only in half of the trials in the $close _{50\%}$ condition. However, the number of errors recorded for such 50% of trials when the obstacle was in place suggests that, also in this regard, the participants of our experiment responded to the $close _{50\%}$ condition similarly to how they did when they were certain about the presence of the obstacle behind them.

The conservative approach by humans to deal with uncertainty under our experimental scenario is also confirmed by the extent and velocity of hand retraction (i.e. $\tilde{x}_{\mathrm{h},\mathrm{max}}$ and $\dot{x}_{\mathrm{h},\mathrm{max}}$), as can be seen in Fig. 3b. The linear mixed-effects analysis revealed significant effects of the interaction between probabilities $\{0\%,50\%,100\%\}$ and obstacle types $\{innocuous,painful\}$) on $\tilde{x}_{\mathrm{h},\mathrm{max}}$ ($\chi ^2(1)=3.89, p<0.05$) and $\dot{x}_{\mathrm{h},\mathrm{max}}$ ($\chi ^2(1)=4.59, p<0.05$). Paired t-tests revealed significant differences on $\tilde{x}_{\mathrm{h},\mathrm{max}}$ for the obstacle type *innocuous*: $\tilde{x}_{\mathrm{h},\mathrm{max}}$ decreases from condition 0 to 100% ($p<0.05$) and from 0 to 50% ($p<0.01$). No significant differences were found on $\dot{x}_{\mathrm{h},\mathrm{max}}$. For the *painful* group, significant differences were found on $\tilde{x}_{\mathrm{h},\mathrm{max}}$, that decreases from condition 0 to 50% $(p<0.01$) and from condition 0 to 100% ($p<0.01$). Significant differences were found concerning $\dot{x}_{\mathrm{h},\mathrm{max}}$ that decreases from condition 0 to 50% ($p<0.05$) and from condition 0 to 100% ($p<0.01$). This means that no significant difference divides the $close _{50\%}$ and the *close* (100%) condition, while significant difference was indeed found between these two conditions and the *away* (0%) condition.

The linear mixed-effects analysis revealed no significant differences on $\varphi (t^*)$, $\theta (t^*)$, $\Delta x_{\mathrm{s}}$ and $\Delta z_{\mathrm{s}}$. However, the distribution of data points in Fig. 4a,b reveal that both sideways displacement of the elbow and its upward movement at the point of maximum hand retraction $\tilde{x}_{\mathrm{h},\mathrm{max}}$, for condition $close _{50\%}$, are comparable with condition *close* (100%), whereas they are considerably different from the values of condition *away* (0%).

Additionally, while the shoulder front and upward movements in relation to the upward movement of the elbow (Fig. 5a,b) did not reveal remarkable differences across the experimental conditions, the slopes of the linear fitted models describing the relationship between values of $\varphi (t^*)$ (lateral elbow displacement) and $\theta (t^*)$ (upward elbow displacement) with hand retraction length (Fig. 4a,b) show that condition $close _{50\%}$ is, also in such regard, comparable with condition *close* (100%).

### Effect of obstacle nature (innocuous vs. painful)

No significant differences in reaction time were found between the two groups (Table 2). Examination of Table 2 reveals that, when the obstacle was placed in the position corresponding to the *midway* condition, subjects collided their elbow more often when the obstacle itself was innocuous (*innocuous* 9.09% vs *painful* 2.78%). The same difference also holds true for the more challenging *close* condition (*innocuous* 25.00% vs *painful* 8.33%).

Analysis of the values of failure ratio for the errors at the hand level (caused by the hand not retracting back enough to avoid impact with the slingshot mechanism) shows that, while the number of failures is comparable in the position corresponding to the *midway* condition (*innocuous* 4.55% vs *painful* 4.17%), such balance shifts when the obstacle is placed in the *close* condition (*innocuous* 2.27% vs *painful* 15.28%). Note that the pain experience elicited by the slingshot mechanism and obstacle were calibrated prior to the start of the experiment to generate comparable perception of pain. Therefore, although not consistently successful, subjects aimed to plan and execute their withdrawal action in a way such as to escape both painful sources. Only in a very small number of cases, subjects made errors at both hand and elbow levels (see second last column of Table 1).

Another effect of placing a painful obstacle can be observed by the analysis of the extent, velocity and acceleration of hand retraction (i.e. $\tilde{x}_{\mathrm{h},\mathrm{max}}$, $\dot{x}_{\mathrm{h},\mathrm{max}}$ and $\ddot{x}_{\mathrm{h},\mathrm{max}}$), as it can be seen in Fig. 3a,b. While the direct comparison—between subjects who were presented with the painful versus the ones who encountered the innocuous obstacle—revealed no significant differences, values of $\tilde{x}_{\mathrm{h},\mathrm{max}}$ show that the hand retracts significantly less when subjects are presented with the painful obstacle, in comparison to the innocuous one. Furthermore, only the group of participants who performed the task in the presence of the painful obstacle shows significant decrease of $\dot{x}_{\mathrm{h},\mathrm{max}}$ and $\ddot{x}_{\mathrm{h},\mathrm{max}}$ as the obstacle gets closer (see section “Effect of obstacle distance”).

The linear mixed-effects analysis showed no significant differences for $\varphi (t^*)$, $\theta (t^*)$, $\Delta x_{\mathrm{s}}$ and $\Delta z_{\mathrm{s}}$. However, the distribution of data points in Fig. 4a,b reveal that introducing a painful obstacle reduces the area of the confidence ellipses that are indicative for the variance of both sideways displacement of the elbow $\varphi (t^*)$ and its upward movement $\theta (t^*)$ at the point of maximum hand retraction $\tilde{x}_{\mathrm{h},\mathrm{max}}$. Such effect is likely to be caused by the lower values of hand retraction in participants who were presented with the painful obstacle, as lower values of $\tilde{x}_{\mathrm{h},\mathrm{max}}$ also require lower displacement of the elbow to succeed in the task. When the painful versus the innocuous obstacle is present, also the areas of the confidence ellipses encapsulating values of front and upward movement of the shoulder at the point $t^*$ of elbow upward displacement is systematically smaller (Fig. 5a,b). This effect is again likely due to the lower values of hand retraction in subjects belonging to the *painful* group, as lower values of $\tilde{x}_{\mathrm{h},\mathrm{max}}$ require lower displacement of the elbow, and thereby less prominent movement of the shoulder to accommodate the overall withdrawal action. Interestingly, smaller variance was recorded in the *away* condition too, although this experimental condition did not differ between the *painful* and *innocuous* group, as no obstacle was present for either group. Such smaller variance could derive from learning effects, i.e. subjects from the *painful* group may have retained—in the *away* trials—part of the motor behavior developed in the trials when the painful obstacle was or could be present.

Ultimately, while analysis of the linear relationship between values of $\varphi (t^*)$ (lateral elbow displacement) with hand retraction length (Fig. 4a), as well as of shoulder front and upward movements in relation to the upward movement of the elbow (Fig. 5a,b) do not reveal systematic differences between the *painful* and *innocuous* groups, it appears for $\theta (t^*)$ (upward elbow displacement) (Fig. 4b) that slopes are steeper in the *painful* group.

## Discussion

Pain signals immediate threats^1^ and triggers relevant motor responses^2^, including the ones elicited by the withdrawal reflex^3^. While the withdrawal reflex^4^—defined as an automatic retraction of an extremity from a noxious stimulus such as heat or pain—was extensively studied, little or no literature has investigated withdrawal actions that start even prior to any physical stimulation, for instance when we visually spot or hear a threatening event. In this exploratory study, we investigated the kinematic characteristics of withdrawal movements of the human upper limb that are triggered by a threatening source at hand level. The experimental task further included an obstacle behind the subject’s arm, positioned at different distances along the direction of withdrawal. Results show that humans do not simply regulate the retraction of the hand alone to avoid both the original threatening source and the collision with the obstacle, but they make use of the degrees of freedom of their upper limb (which can be considered as 12 DoF serial kinematics)^5,6^—in a highly coordinated manner. Specifically, in the presence of an obstacle placed behind us, not only do we stop our rapid withdrawal action earlier than what we would do in the absence of the obstacle, but we also complement this action by bending the elbow sideways, keeping it away from collision. While some participants bend their elbow to the inside and retract the hand less, others opt for longer spans of hand retraction, but bend their elbow to the outside, where they can count on a more abundant range of possible displacement. Therefore, different subjects use distinct motor strategies^7,8^. Subjects tend to stick to bending their elbow either to the right of, to the left of, or along the longitudinal axis of the hand, across trials and conditions. To accommodate such withdrawal action, we raise the elbow and move the shoulder forward and upwards—with such coordinated movement more accentuated for the subjects who bend their elbow to the outside (i.e. with greater hand retraction and lateral elbow displacement). The closer the obstacle, the more discernible this behavior appears. It is likely that the overall withdrawal movement originates from a highly coordinated action between hand, elbow and shoulder^9^, although the shoulder movement could also be due to the physical constraint placed at hand level (i.e. its fixation to the slider). Another interesting interpretation is that the subjects may utilize the mechanical coupling of the upper limb’s joints to accelerate the hand, specifically by using the inertia of the shoulder, whereby the shoulder is “thrown” forward and upwards in order to facilitate acceleration of the hand, to keep it away from the slingshot range.

Retraction movements have been studied from the traditional perspective of motor control, with sensory information together with self-intention leading to a motor action via a planning process^10^. The overall success demonstrated by our participants to deal with a task that requires a prompt and explosive reaction shows how such complex action needs to be planned in advance. While the planning phase is fundamental in similar tasks, continuously shaping the withdrawal trajectory during the task is also crucial, with results from a study that investigated object throwing^11^ suggesting that creating a timing window for determining tolerance for variability, and consequently accuracy, requires shaping the arm trajectory not only before, but even after the critical release moment.

To investigate whether and how the withdrawal strategy changes when humans are subject to uncertainty, we introduced a condition where participants were informed that the obstacle may or may not be present behind them. They were made explicitly aware that the chance corresponded to a 50% probability. Specifically, results were examined to ascertain whether humans adopt a conservative strategy and plan the movement according to the worst-case scenario (i.e. *close* condition), or they rather take risks and behave more similarly to the *away* condition, in the attempt to save the mental workload required for planning a more constrained, demanding movement. Mental workload was recently defined to include perceptual, motor and also cognitive components^12^, with anticipation of cognitive effort relying on the cortico-limbic network via activation in the anterior cingulate cortex and the striatum^13^. In particular, we expected results to reveal a relatively more conservative strategy for participants who were possibly presented with a painful obstacle behind them. Anticipation of pain, i.e. the activation of mechanisms to prevent future harm by learning to recognize signals of impending pain^14^ prepares the organism for a potentially painful outcome, as pain can be suppressed or enhanced by factors such as immediate threat and predicted reward^15,16^. While these physiological studies have explored the brain neural systems underlying pain, and motivation, the experiment of this present work has investigated the effect of pain anticipation on subjects’ decision-making from a behavioral standpoint. In this, our findings reveal that humans always adopt a conservative strategy and plan the movement according to the worst-case scenario, regardless of the painful or innocuous nature of the obstacle. In contrast, a previous study^17^ investigating the effect of pain probability on human motor behavior during an exploration task showed that the explicit probability that subjects may encounter an object does not influence their approach speed when the object is innocuous, whereas the probability of encountering a noxious object decreases their approach velocity. Crucially, it could be observed that the speed of the reaching movement decreased gradually as the probability that the participants could encounter a noxious object increased, in a trade-off between safety and time to complete the task. The difference between the results from these two studies could be explained by the intrinsic task motivation in our present experiment, where the explicit goal to avoid impact with the obstacle could have by far exceeded our subjects’ will to modulate their motor behavior efficiently. Furthermore, although subjects could plan the movement in advance, the impulsive and challenging nature of the motor task itself could have allowed little cognitive resources for developing a trade-off like behavior.

Such a conservative approach was also likely to prevent participants from making more errors under the condition of uncertainty, especially when the obstacle behind was painful. While pain anticipation is generally associated with stress and correlates with the degree of uncertainty^18^—and motor performance is known to depend on the level of stress according to an inverse-U relationship^19^—the same error ratio was recorded between the uncertain and the certain *close* condition.

The effect of introducing a painful obstacle was systematically studied by assigning the participants of our experiment to two different groups, one of which executed the experiment with an innocuous obstacle and the other one with a painful one, as in the latter case collision triggered electrical stimulation. This painful event was meant to introduce an additional layer of motivation. While subjects from both groups *innocuous* and *painful* were asked to avoid impact with the obstacle behind them (i.e. they were motivated by the definition of the task goal itself), the group *painful* was additionally subject to the motivation of avoiding the electrical pain. We found that, when the impact with the obstacle is painful, success rate at elbow level increases. This is achieved by controlling retraction more carefully, i.e. decreasing velocity and acceleration of hand retraction. Furthermore, we modulate the elbow movement (i.e. we raise the elbow more for the same extent of hand retraction) such that it is as far away as possible from the obstacle. However, such strategy increases the chance to fail at hand level. The increased failure ratio at the level of the hand for the *close* condition—only for the group subject to the painful obstacle—is likely to be due to the attempt by participants to minimize hand retraction, thus avoiding collision with the painful obstacle. This strategy resulted in over-minimization of hand retraction, thereby exposing them to the impact with the slingshot mechanism.

These results indicate that, in contrast to what it could be concluded by observing group *innocuous* alone, the twofold objective of avoiding both hand- and elbow-level collisions could not be decoupled in the present task. Therefore, it is unlikely that a subset of joints and movement range were primarily responsible for the completion of one and the other goal, as is used to control complex robotic systems^20,21^. Reducing the number of elbow collisions at the cost of hand collisions could underlie hierarchical processes^22^, on the basis of which participants switched from one motor strategy to another, given the perceived consequence of the immediate threats (i.e. pain at the hand versus elbow). Although the neural correlates of pain and its effect on motor behaviour under the scenarios presented to our subjects can only be reliably identified with purposely designed versions of our experiment, literature suggests that neural control of movement of higher vertebrates is organized into a distributed set of structures that are both anatomically and functionally hierarchical^22^. Withdrawal from noxious threats could be mainly driven by low-level controllers that function autonomously, whereas the switch from one strategy to another could be regulated by higher-level control layers^23,24^, and mediated by the activity of sub-cortical structures such as the basal ganglia, which have been proposed as motor program selectors among different modes of coordinated behavior^25^.

In summary, we found that humans—when aware of the incoming threat—plan the retraction movement and make use of the degrees of freedom of their upper limb in a highly coordinated manner, adopting a conservative approach. Furthermore, introducing pain at the elbow level as an extra layer of motivation does modify behavior. Specifically, the tendency by subjects to reduce elbow collisions at the cost of hand collisions reveals our inability to decouple the motor task, and underlies hierarchical processes. Such results further reveal how humans prevent damage, pain and injury^2,17^, adding to our fundamental understanding of motor control. Possibly, they will give insight into the implementation of artificial pain mechanisms for robots^26^, serving as reference to inspire the design of safe robotic^27^ and prosthetic^28^ retraction movements.

## Methods

### Participants

The experiments took place at the Institute of Automatic Control of Gottfried Wilhelm Leibniz Universität Hannover (LUH). The study was approved by the ethics committee of LUH, and all experiments were conducted according to the principles in the Declaration of Helsinki. Each of the 23 subjects gave their written informed consent prior to participating in the study. The subjects were all male and right-handed (as was assessed using the Edinburgh Handedness Inventory^29^), and no subject had a known neuromuscular disorder or recent injury at arm level. All subjects had normal or corrected to normal vision.

A group of 11 subjects (aged $27.36\pm 2.29\ \hbox {years}$) used their dominant arm to perform a withdrawal task, escaping a slap on their hand while avoiding a collision between their elbow and an *innocuous* obstacle. A second group of 12 subjects (aged $26.5\pm 4.5\ \hbox {years}$) performed the same task under the same conditions while contact with the obstacle at elbow level was *painful*.

### Experimental setup and sequence

The experiment took place in a sound-isolated and electromagnetically shielded measuring chamber. Figure 1b shows sketches of the experimental setup and sequence. The subject is seated comfortably on a chair and remains in an upright position during the experiment. In order to reduce complexity, the hand is fixed on a one DoF *slider* mechanism, i.e. a moving plate on a rail that is orthogonal to the coronal plane. Above the hand is a preloaded *slingshot*, a flexible sheet metal (Fig. 1b $\textcircled {1}$) retained by an electromagnet. An acoustic signal—aired 500 ms before the electromagnet is switched off (Fig. 1b $\textcircled {2}$)—warns the subject, who is prompted to retract the hand and so avoid the impact with the sheet metal (Fig. 1b $\textcircled {3}$).

An innocuous or painful *obstacle* is placed behind the elbow. This thin and flexible metallic panel will not harm a subject accidentally hitting it with the elbow while withdrawing the hand to avoid the slap. The goal for all subjects (Fig. 1b $\textcircled {4}$) is to withdraw the hand in such a way that they can (1) escape the mechanical pain threat by the slingshot mechanism by retracting their elbow and (2) also avoid collision with the obstacle. For subjects in the *painful* group, an electrical stimulation is released to their elbow when they touch the panel.

### Apparatus

**Figure 6:**
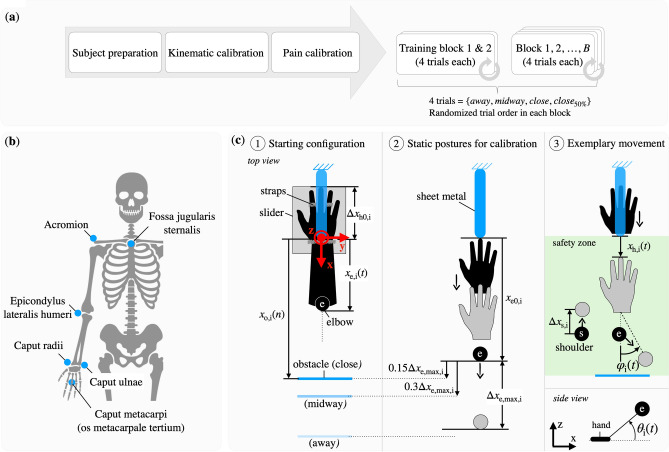
(**a**) Experimental protocol sequence. (**b**) Marker placement. (**c**) Measurements for data analysis of subject *i*.

A real-time measurement computer was used together with a *National Instrument 9144 EtherCAT chassis* to control and synchronize the slingshot mechanism, a motion tracking system, and an electrical stimulator device. A *Vicon MXT10s* (Vicon Motion Systems Ltd, UK) system with eight infrared cameras tracking positions of passive reflected markers (15 mm diameter) at 500 Hz was used to capture human upper limb motions in Cartesian space. Six *essential* markers were placed on significant anatomical landmarks: *Os metacarpale tertium (Caput metacarpi)*, *Caput ulnae*, *Caput radii*, *Epicondylus lateralis humeri*, *Acromion* and *Fossa jugularis sternalis* as shown in (Fig. 6b). Note that the marker on the *Fossa jugularis sternalis* remains unused in this paper, but can be employed in e.g. future analysis based on human musculoskeletal models^5^.

For the *painful* group of participants, a computer controlled *Single Constant Current Stimulator* of *Dantec*$^{\mathrm{TM}}$ *Keypoint* *G4 Workstation* (Natus Medical Incorporated, USA) was used to percutaneuosly stimulate the *Nervus cutaneus brachii posterior*, a sensory branch of the *Nervus radialis*, through a 2-point-bipolar electrode placed three finger widths above the *Humerus coronoid fossa* on the dorsal side of the upper limb. A single burst consisting of ten rectangular pulses with 0.2 ms duration at 300 Hz is applied when the elbow contacts the obstacle, as detected by the experimenter following a visually-unequivocal vibration by the thin metallic panel.

### Experimental protocol

Figure 6a shows the protocol sequence. Each subject is asked to sit comfortably on a chair and keep the back in an upright position during the experiment. After motion tracking markers (and also the electrical stimulator for the *painful* group of subjects) are positioned accurately, the hand is fixed to the slider mechanism using straps tightened at the wrist, ring finger and index finger (Fig. 6c $\textcircled {1}$). A calibration procedure is then performed to determine (1) the starting position of the hand and (2) the distance between the obstacle and the elbow (see Sect. Kinematic calibration). Furthermore, (3) a pain calibration is conducted so that the level of pain from hand slap is normalized across all subjects (see Sect. Pain calibration). A complementary calibration protocol is undertaken by the *painful* group of participants in order to ensure a comparable perception level when hitting the obstacle with the elbow as when the hand is slapped (see Sect. Pain calibration). Before the experiment started, all subjects conducted two training blocks of 4 trials each to get used to the system and to minimize in-study learning effects (Fig. 6a).

For the *painful* group of subjects, the experiment consisted of $N=24$ trials divided in $B=6$ blocks. For the *innocuous* group it consisted of $N=16$ trials divided in $B=4$ blocks (Fig. 6a). After having completed the experiments with the *innocuous* group, we decided to increase the total number of trials for the *painful* group as we judged that the *innocuous* experiment duration was not altering the quality of data, i.e. did not cause fatigue to subjects nor affected their attention. Each block had 4 trials with conditions $\{away, midway, close, close_{50\%}\}$, where these denominations correspond to the respective distance between the elbow position at the start of the trial and the position of the obstacle (Fig. 6c $\textcircled {1}$). The conditions *close* and *midway* could be used to investigate how subjects deal with a challenging versus moderate difficulty level, relative to the *away* condition without obstacle. In the $close _{50\%}$ condition, participants were informed that there would be an obstacle in 50% of the cases. The trials in each block were randomized to minimize any bias and in-study time effects (Fig. 6a). However, the same random sequence of trials was used for all subjects.

The subjects wear blinkers during the experiment in order to avoid visual feedback of the obstacle. They are informed of which specific condition would be presented before each trial and could (haptically) explore the surroundings at their back and locate the obstacle in order to plan the withdrawal movement. Subjects are then asked to reach the starting position and tell the experimenter when they were ready. Any time during 30 s after a vocal “Start” cue, an acoustic signal is emitted, 500 ms after which the slingshot mechanism would attempt to slap the subject’s hand.

### Kinematic calibration

For each participant, the starting position of the hand $x_{\mathrm{h},\mathrm{i}}(t=0)= \Delta x_{\mathrm{h}0,\mathrm{i}}$ is selected so that the dorsum can be hit by the sheet metal, adjacent to the wrist strap (Fig. 6c $\textcircled {1}$). To ensure repeatability across trials, the position is marked by a screw on the slider rail. In order to determine the individual obstacle distances *away*, *midway* and *close*, each subject is asked to place the hand in the first position along the longitudinal axis of the sheet metal, such that the sheet metal itself no longer reaches the hand (Fig. 6c $\textcircled {2}$), keeping the elbow aligned to the same axis (i.e. $\varphi _{\mathrm{i}}\approx 0^{\circ }$). In this static configuration, we define $x_{\mathrm{h},\mathrm{i}}:=0\,\mathrm{m}$ and measure the position of the elbow $x_{\mathrm{e}0,\mathrm{i}}$. The maximum elbow displacement $x_{\mathrm{e},\mathrm{max},\mathrm{i}}=x_{\mathrm{e}0,\mathrm{i}}+\Delta x_{\mathrm{e},\mathrm{max},\mathrm{i}}$ is then identified by asking the subject to simulate the withdrawal movement, maximizing the elbow travel and maintaining $\varphi _{\mathrm{i}}\approx 0^{\circ }$.

### Pain calibration

Pain underlies sensory–discriminatory, cognitive–evaluative and affective-motivational processes^30^, and is by definition subjective^31^. While it is not possible to measure pain objectively^32^, efforts have been made to develop reliable methods and tools^33^ to estimate it. In this context, we have included a pain calibration procedure with the aim to remove high-granular bias in our exploratory study.

In order to normalize the pain intensity caused by the slingshot across all subjects, we used a verbal *Visual Analogue Scale* (VAS) of 100 mm length, graded by *no pain* (0–4 mm), *mild pain* (5–44 mm), *moderate pain* (45–74 mm) and *severe pain* (75–100 mm)^34^. The subject is asked to place the *distal interphalangeal joint* of the middle finger underneath the slingshot. Three paddings of varying thickness $\{2,3,4\}\,\mathrm{mm}$ are attached to the sheet metal starting with 4 mm. Then, the slingshot is activated and the subject rates the perceived pain on the VAS. The padding whose VAS value is closest to the middle of the scale is then chosen.

To verify that hitting the obstacle does not harm the subjects, the obstacle is set at $x_{\mathrm{e}0,\mathrm{i}}$ and each subject is asked to withdraw the elbow and hit the obstacle first slowly and then at increasing speeds. The VAS is again used to confirm that the impacts are harmless.

A complementary calibration is undertaken by the *painful* group of participants in order to ensure comparable perception levels of pain between the electrical stimulation at the elbow and the hand slap. A staircase method^35^ is used to calibrate the pain stimulation. First, the sensory threshold (ST) is determined by gradually increasing the current in steps of 0.1 mA (starting from 0 mA) until the subject notices the stimulus. Then, the *pain threshold* (PT) is determined by further increasing the current until the subject perceives the stimulus as painful. To verify the PT, the current is then increased to around 1 mA and gradually decreased afterwards till the subject marks the intensity as innocuous.

In order to relate the pain experienced at the elbow (caused by the *electrical* stimulus) to the one at the hand (caused by a *mechanical* stimulus), the intensity of the electrical stimulus is then subsequently increased times PT, until the participant reports that the resulting pain perception matches with the one induced by the slingshot mechanism at the hand.

### Measures

The normalized *hand retraction* for each trial is defined as

1 $$\begin{aligned} \tilde{x}_{\mathrm{h},\mathrm{max}}:= \frac{x_{\mathrm{h}}(t^*)}{|\Delta x_{\mathrm{h}0}|}, \end{aligned}$$

where $t^{*}$ is the time instant when $x_{\mathrm{h}}(t)$ reaches its maximum retraction during the withdrawal movement (Fig. 6c $\textcircled {3}$). The maximum hand retraction $x_{\mathrm{h}}(t^*)$ is normalized to the individual distance $|\Delta x_{\mathrm{h}0}|$. Such distance corresponds to the span of retraction necessary for subjects to exit the slingshot impact range with their hand (Fig. 6c $\textcircled {1}$). This normalization yields an intuitive way to interpret the extent of the hand retraction length. For example, $\tilde{x}_{\mathrm{h},\mathrm{max}}>1$ means that the hand successfully escaped impact with the slingshot mechanism. In the analysis we also considered the maximum velocity $\dot{x}_{\mathrm{h},\mathrm{max}}$ and acceleration $\ddot{x}_{\mathrm{h},\mathrm{max}}$ of hand retraction.

The *reaction time* is defined as

2 $$\begin{aligned} \Delta t_{\mathrm{r}}:= t' - t_{\mathrm{beep}}, \end{aligned}$$

where $t_{\mathrm{beep}}$ is the time instant when the acoustic signal starts to ring and $t'$ is the time instant when the Cartesian acceleration of the hand marker *Os metacarpale tertium (Caput metacarpi)* reaches $0.1\,\hbox {m}/\hbox {s}^{2}$. This threshold, determined empirically, was introduced to filter out any hand movements taking place prior to the withdrawal action.

Furthermore, we define the *failure ratio* as

3 $$\begin{aligned} f := \frac{N_{\mathrm{failed}}}{N_{\mathrm{total}}} 100\%, \end{aligned}$$

where $N_{\mathrm{total}}$, $N_{\mathrm{failed}} \in {\mathbb {N}}^+$ denote, respectively, the total number of trials and the total number of failed trials within a group $\{innocuous, painful\}$, across a condition $\{away, midway, close, close_{50\%}\}$.

Other measures appearing in the results are the *elbow angles* $\varphi (t^*)$, $\theta (t^*)$, and *Cartesian shoulder positions* (Fig. 6c $\textcircled {3}$)

4 $$\begin{aligned} \Delta x_{\mathrm{s}}&= x_{\mathrm{s}}(t') - x_{\mathrm{s}}(t^*), \end{aligned}$$

5 $$\begin{aligned} \Delta z_{\mathrm{s}}&= z_{\mathrm{s}}(t') - z_{\mathrm{s}}(t^*). \end{aligned}$$

### Data analysis

The data of recorded measures were analyzed using a linear mixed-effects model^36^

6 $$\begin{aligned} r_{\mathrm{ij}}=\begin{bmatrix} 1 \\ a_{1,\mathrm{ij}} \\ a_{2,\mathrm{ij}} \\ a_{1,\mathrm{ij}}a_{2,\mathrm{ij}} \end{bmatrix}^T \begin{bmatrix} \alpha _0 \\ \alpha _1 \\ \alpha _2 \\ \alpha _3 \end{bmatrix} +\begin{bmatrix} 1&a_{1,\mathrm{ij}} \end{bmatrix} \begin{bmatrix} \beta _{0,\mathrm{j}} \\ \beta _{1,\mathrm{j}} \end{bmatrix} + \epsilon _{\mathrm{ij}}, \end{aligned}$$

where subscript $j=1,\ldots ,N$ denotes the observation (i.e. one for each trial) for subject *i*. As fixed effects we introduced an intercept, two explanatory variables $a_{1,\mathrm{ij}}$ and $a_{2,\mathrm{ij}}$, and the interaction $a_{1,\mathrm{ij}}a_{2,\mathrm{ij}}$. Parameter $\beta _{0,\mathrm{j}}$ denotes the random effect for the by-subject intercepts and $\beta _{1,\mathrm{j}}$ is the random effect for by-subject slopes for the effect of $a_{1,\mathrm {ij}}$.

In order to understand the effect of varying *obstacle distance* on the subject’s behavior, we applied the model in (6) with explanatory variables $a_{1,\mathrm{ij}} \in \{away,midway,close\}$ and $a_{2,\mathrm{ij}} \in \{innocuous,painful\}$ on maximum hand position $\tilde{x}_{\mathrm{h},\mathrm{max}}$ (1), $\dot{x}_{\mathrm{h},\mathrm{max}}$, $\ddot{x}_{\mathrm{h},\mathrm{max}}$, $\varphi (t^*)$, $\theta (t^*)$, $\Delta x_{\mathrm{s}}$ (4), $\Delta z_{\mathrm{s}}$ (5) and reaction time $\Delta t_{\mathrm{r}}$ (2). Pairwise comparisons (paired t-tests) with Bonferroni corrections were performed to compare average measures within each group $\{innocuous,painful\}$ across the different obstacle distance $\{away,midway,close\}$.

In order to understand the effect of varying *obstacle presence uncertainty* on the subject’s behavior, we applied the model in (6) with explanatory variables $a_{1,\mathrm{ij}} \in \{0\%(away),50\%(close_{50\%}),100\%(close)\}$ and $a_{2,\mathrm{ij}} \in \{innocuous,painful\}$ on maximum hand position $\tilde{x}_{\mathrm{h},\mathrm{max}}$ (1), $\dot{x}_{\mathrm{h},\mathrm{max}}$, $\ddot{x}_{\mathrm{h},\mathrm{max}}$, $\varphi (t^*)$, $\theta (t^*)$, $\Delta x_{\mathrm{s}}$ (4), $\Delta z_{\mathrm{s}}$(5) and reaction time $\Delta t_{\mathrm{r}}$ (2). Pairwise comparisons (paired t-tests) with Bonferroni corrections were performed to compare average measures within each group $\{innocuous,painful\}$ across the different obstacle presence probabilities $\{0\%,50\%,100\%\}$.

Following the application of those linear mixed-effects models, in order to understand the effect of varying *obstacle nature*, single t-tests were performed over distances $\{away,midway,close\}$ between groups $\{innocuous,painful\}$ and over probabilities $\{0\%,50\%,100\%\}$ between groups $\{innocuous,painful\}$ for values of $\tilde{x}_{\mathrm{h},\mathrm{max}}$, $\dot{x}_{\mathrm{h},\mathrm{max}}$ and $\ddot{x}_{\mathrm{h},\mathrm{max}}$. Bonferroni corrections were performed here as well.

For all linear mixed-effects models, no obvious deviation from homoscedasticity and normality was observed from the inspection of residual plots and the p-values were obtained by likelihood ratio tests of the full model with the effect in question against the model without the effect in question^37^. A 5% significance level was used in all tests.
